# O-GlcNAc Modification Is a Promising Therapeutic Target for Diabetic Retinopathy

**DOI:** 10.3390/ijms25116286

**Published:** 2024-06-06

**Authors:** Wenkang Dong, Laraib Imdad, Shengnan Xu, Yinli Wang, Chengzhi Liu, Shiyu Song, Zechuan Li, Ying Kong, Li Kong, Xiang Ren

**Affiliations:** 1 Department of Histology and Embryology, College of Basic Medicine, Dalian Medical University, Dalian 116044, China; dwksq1997@163.com (W.D.); laraibimdad@hotmail.com (L.I.); xsn18246699163@163.com (S.X.); wyl382306126@163.com (Y.W.); cheng.zhi.liu@outlook.com (C.L.); ssyashley@163.com (S.S.); vnumqfen@outlook.com (Z.L.); kongli@dmu.edu.cn (L.K.); 2 Key Laboratory of Reproductive and Developmental Biology, Dalian Medical University, Dalian 116044, China; 3 Core Laboratory of Glycobiology and Glycoengineering, College of Basic Medical Sciences, Dalian Medical University, Dalian 116044, China

**Keywords:** diabetic retinopathy, O-GlcNAc modification, photoreceptor, apoptosis, AMPK

## Abstract

Diabetic retinopathy (DR) is a very serious diabetes complication. Changes in the O-linked N-acetylglucosamine (O-GlcNAc) modification are associated with many diseases. However, its role in DR is not fully understood. In this research, we explored the effect of O-GlcNAc modification regulation by activating AMP-activated protein kinase (AMPK) in DR, providing some evidence for clinical DR treatment in the future. Bioinformatics was used to make predictions from the database, which were validated using the serum samples of diabetic patients. As an in vivo model, diabetic mice were induced using streptozotocin (STZ) injection with/without an AMPK agonist (metformin) or an AMPK inhibitor (compound C) treatment. Electroretinogram (ERG) and H&E staining were used to evaluate the retinal functional and morphological changes. In vitro, 661 w cells were exposed to high-glucose conditions, with or without metformin treatment. Apoptosis was evaluated using TUNEL staining. The protein expression was detected using Western blot and immunofluorescence staining. The angiogenesis ability was detected using a tube formation assay. The levels of O-GlcNAc transferase (OGT) and O-GlcNAcase (OGA) in the serum changed in the DR patients in the clinic. In the diabetic mice, the ERG wave amplitude and retinal thickness decreased. In vitro, the apoptotic cell percentage and Bax expression were increased, and Bcl2 expression was decreased in the 661 w cells under high-glucose conditions. The O-GlcNAc modification was increased in DR. In addition, the expression of GFAT/TXNIP O-GlcNAc was also increased in the 661 w cells after the high-glucose treatment. Additionally, the Co-immunoprecipitation(CO-IP) results show that TXNIP interacted with the O-GlcNAc modification. However, AMPK activation ameliorated this effect. We also found that silencing the AMPKα1 subunit reversed this process. In addition, the conditioned medium of the 661 w cells may have affected the tube formation in vitro. Taken together, O-GlcNAc modification was increased in DR with photoreceptor cell degeneration and neovascularization; however, it was reversed after activating AMPK. The underlying mechanism is linked to the GFAT/TXNIP-O-GlcNAc modification signaling axis. Therefore, the AMPKα1 subunit plays a vital role in the process.

## 1. Introduction

Diabetes mellitus (DM) is a metabolic disease characterized by high blood glucose. The latest statistics from the International Diabetes Federation (IDF) show that the global prevalence of diabetes among those between 20 and 79 years of age is estimated to increase from 10.5% (536.6 million people) in 2021 to 12.2% (783.2 million) in 2045 [1]. The different complications caused by DM have become the main reasons for death in diabetic patients [2], including complications of the eyes, heart, and kidneys [3].

Diabetic retinopathy (DR) is the main cause of irreversible visual impairment and blindness, which seriously affects people’s quality of life [4]. DR has been widely discussed as one of the most serious diabetes complications for a long time. Historically, researchers thought DR only manifested as microangiopathic lesions, which led to the loss of vision in DR patients. Therefore, DR has been clinically defined as a microvascular disease for many years [5]. However, there is the growing evidence that retinal neurodegeneration, as a significant mechanism of injury in the development of DR, precedes clinically detectable microvascular injury [6,7,8,9]. Moreover, increasing evidence indicates that retinal neurodegeneration and microangiopathy participate in the pathogenesis of DR together [10]. Additionally, in our previous research, we found that decreasing 661 w cell apoptosis under high glucose conditions, which helps maintain photoreceptor cell structure, function stability, and micro-environment hemostasis, can delay DR in vitro [11]. Therefore, targeting photoreceptor cells for neurodegeneration prevention is a promising method for delaying DR at an early clinical stage in the future.

There are many factors leading to DR development, such as the production of glycosylation end products (AGES) [4,12], the accumulated generation of reactive oxygen species (ROS) [5,13], polyol pathway flux increase, protein kinase C pathway activation [14], and upregulation of the hexosamine pathway, leading to O-linked N-acetylglucosamine (O-GlcNAc) modification [15], by which serine or threonine moieties are attached to a single O-linked N-acetylglucosamine. A unique type of post-translational modification (PTM) occurs on proteins both in the cytoplasm and nucleus. O-GlcNAc modification is regulated by O-GlcNAc transferase (OGT) and O-GlcNAcase (OGA), and it plays a direct role in the formation of neurodegenerative lesions [16]. It has been shown that dysregulated O-GlcNAc modification is associated with many diseases, including obesity [17], Alzheimer’s disease [18], cancer [19], and diabetes [20]. Moreover, some studies have suggested that O-GlcNAc modification is also associated with several pathological disease processes with hyperglycemic phenotypes, especially diabetes [21]. However, few studies have focused on the role and effect of O-GlcNAc modification regulation in DR. Therefore, it is important to explore and find a possible way to regulate O-GlcNAc modification and elucidate its related mechanism.

AMP-activated protein kinase (AMPK) is an important energy-sensing molecule in cells and is involved in many biological processes, such as lipid metabolism [22], the maintenance of mitochondrial homeostasis [23], and glycolytic processes [24]. It has received much attention as a potential target for the treatment of metabolism-related diseases, including diabetes [25], obesity [26], and cancer [27]. However, the effect of AMPK activation on O-GlcNAcylation modification in the process of DR is not fully understood.

Therefore, in the present study, a diabetic mouse model was established, and 661 w cells underwent high-glucose treatment to investigate the role of O-GlcNAc modification in the process of DR and to explore the effects of AMPK activation on this process and related mechanisms, with the aim of finding new therapeutic targets to delay the development of diabetic retinopathy.

## 2. Results

### 2.1. OGT and OGA Expression Changes Related to Diabetic Retinopathy

**Figure 1:**
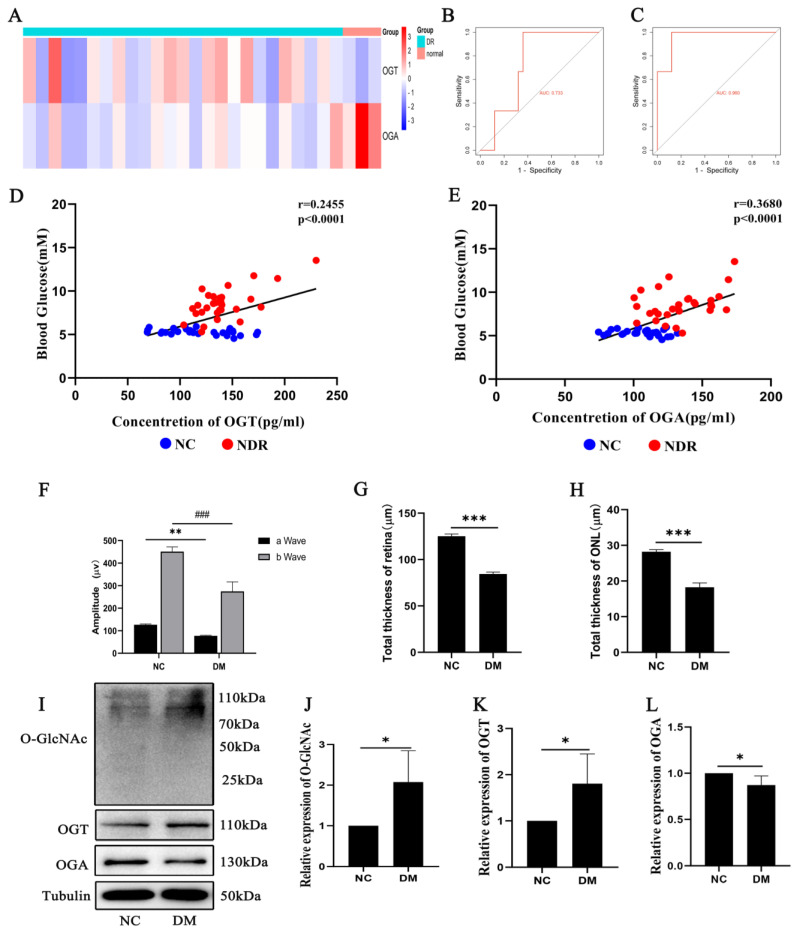
The OGT and OGA expression changes are related to diabetic retinopathy. (**A**) Heat map visualizing the differential expressions of OGT and OGA genes. The receiver operating characteristic (ROC) curve was used to validate the diagnostic utility of OGT (**B**) and OGA (**C**). Analysis of the correlation between the blood glucose level and the concentration of OGT (**D**) and OGA (**E**). (**F**) Retinal morphology changes observed by HE staining in diabetic mice. (**G**) The total retina thickness was measured in diabetic mice. (**H**) The outer nuclear layer thickness was measured in diabetic mice. The expressions of O-GlcNAc (**I**,**J**), OGT (**I**,**K**), and OGA (**I**,**L**) were detected using Western blot in diabetic mice. The differences between the two groups were determined using a t-test. The data are expressed as the mean ± SD (The data were obtained from the mice, *n* = 3 for each group). * *p* < 0.05; ** *p* < 0.01; *** or ^###^ *p* < 0.001.

Figure 1A shows the biological information of the samples selected from the GEO database (GSE102485 dataset; https://www.ncbi.nlm.nih.gov/geo/, accessed on 12 February 2023), with differential expressions of OGT and OGA in the same samples from different groups. The OGT expression was upregulated, and the OGA expression was downregulated in the DR patients compared to the normal controls. The receiver operating characteristic (ROC) curve validation results show that the area under curves (AUCs) for OGT and OGA were 0.733 and 0.960, respectively, which predicted their clinical diagnostic value (Figure 1B,C). The ELISA data of the clinical blood samples and their correlation analysis with blood glucose show that both the OGT and OGA levels in the serum were altered in the patients at an early stage of DR compared to normal people and correlated with the blood glucose levels (Figure 1D,E).

We also did related research on diabetic mice. The ERG results show that the peak values of the a wave and b wave were decreased in the DM group compared to the negative control (NC) group (*p* < 0.01, *p* < 0.001) (Figure 1F). Moreover, morphological changes were observed by HE staining and measuring the total thicknesses of the retina and outer nuclear layer (ONL). The total thicknesses of the retina and ONL were decreased in the DM group compared to the NC group (*p* < 0.001) (Figure 1G,H). Additionally, the expressions of O-GlcNAc and OGT were increased, and the expression of OGA was decreased in the DM group compared to the NC group (*p* < 0.05) (Figure 1I–L). This suggests that photoreceptor cell damage is related to changes in the O-GlcNAc modification in diabetic retinopathy.

### 2.2. Effects of AMPK Activation on O-GlcNAc Modification Changes That Lead to Photoreceptor Cell Damage in Diabetic Retinopathy

**Figure 2:**
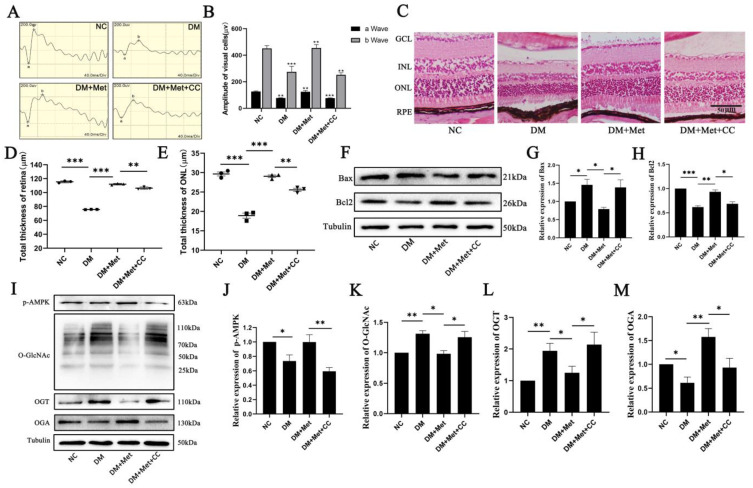
The effects of AMPK activation on O-GlcNAc modification changes that lead to photoreceptor cell damage in diabetic retinopathy. (**A**) Diabetic mice’s retinal function was measured using ERG. The letter a represented the peak of a wave and the letter b represented the peak of b wave. (**B**) The peak values of the a wave and b wave of ERG in diabetic mice. (**C**) Retinal morphological changes in diabetic mice were observed using HE staining. The scale bar is 50 μm. (**D**) The total retina thickness was measured in diabetic mice. (**E**) The outer nuclear layer thickness was measured in diabetic mice. The triangles, circles, squares represented the data showed in the different groups. (**F**) Western blot was used to detect the apoptosis-associated protein Bax/Bcl2 expression in diabetic mice. (**G**) Western blot band analysis of Bax. (**H**) Western blot band analysis of Bcl2. (**I**) The bands of p-AMPK and O-GlcNAc modification were detected using Western blot in diabetic mice. Western blot band analysis of p-AMPK (**J**), O-GlcNAc (**K**), OGT (**L**), and OGA (**M**). The differences between the various groups were determined using a one-way analysis of variance. The data are expressed as the mean ± SD (*n* = 3 for each group). * *p* < 0.05; ** *p* < 0.01; *** *p* < 0.001.

In Figure 2A,B, the ERG results in the diabetic mice showed that the peak values of the a wave and b wave were lower in the DM group compared to the NC group (*p* < 0.01, *p* < 0.001). However, this was reversed after AMPK activation (metformin treatment) or inhibition (metformin and compound C treatment) in diabetic mice (*p* < 0.01). Moreover, the total retina thickness and ONL thickness in diabetic mice were decreased in the DM group compared to the NC group (*p* < 0.001), which was also reversed after AMPK activation (*p* < 0.001) (Figure 2C–E). However, the thickness of the retina and ONL were also partially reduced after compound C treatment, which might be partially involved in this process (*p* < 0.01). We also used Western blot to evaluate the cell apoptosis related protein expression in the retinas of the diabetic mice. The results show that the Bax expression was increased and the Bcl2 expression was decreased in the DM group compared to the NC group (*p* < 0.05), which was reversed after AMPK activation or inhibition in the diabetic mice (*p* < 0.01) (Figure 2E–G). Furthermore, the O-GlcNAc modification changes were also evaluated using Western blots. The results show that the O-GlcNAc expression and OGT expression were increased, and the OGA expression was decreased in the DM group compared to the NC group (*p* < 0.05, *p* < 0.01). However, this process was reversed in the diabetic mice after activation using metformin (DM + Met) or after the inhibition of AMPK using compound C (DM + Met + CC) (*p* < 0.01) (Figure 2H–L). These results suggest that photoreceptor cell damage is associated with O-GlcNAc modification changes that AMPK activation could regulate in diabetic retinopathy.

### 2.3. Photoreceptor Cell Damage In Vitro Is Related to O-GlcNAc Modification Changes after High Glucose Treatment

**Figure 3:**
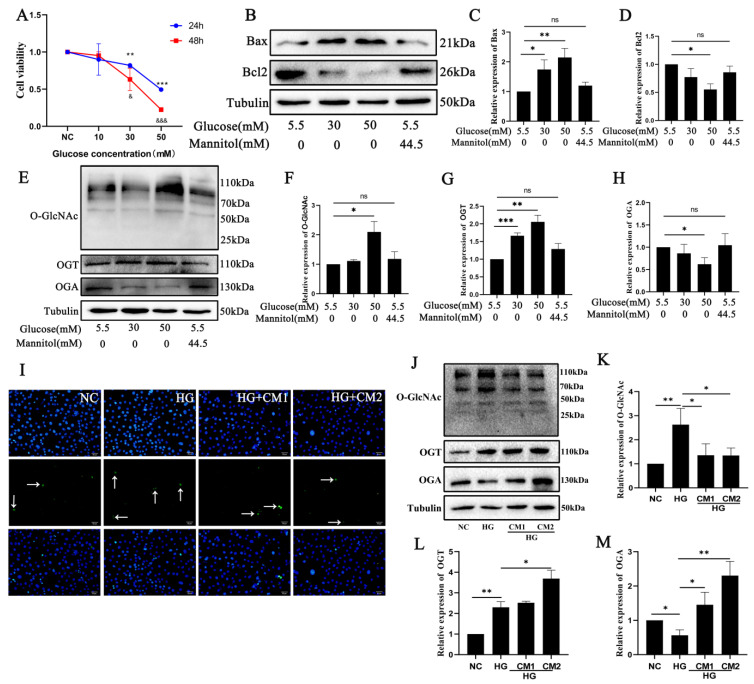
Diabetes-induced photoreceptor cell damage is related to O-GlcNAc modification changes in vitro. (**A**) The effect of glucose treatment on 661 w cell viability. (**B**) The expression of apoptosis-related proteins Bax and Bcl2 in 661 w cells was detected using Western blot after high-glucose treatment. (**C**) Western blot band analysis of Bax. (**D**) Western blot band analysis of Bcl2. (**E**) O-GlcNAc modification expression in 661 w cells was detected using Western blot after high-glucose treatment. Western blot band analysis of O-GlcNAc (**F**), OGT (**G**), and OGA (**H**). (**I**) Effects of conditioned medium from Müller cells on high-glucose-induced apoptosis in 661 w cells detected using TUNEL staining. The arrows pointed the positive cells. The scale bar is 50 μm. (**J**) Western blot was used to detect O-GlcNAc modification changes in 661 w cells after conditioned medium from Müller cell treatment under high-glucose conditions. Statistical analysis of the expressions of O-GlcNAc (**K**), OGT (**L**), and OGA (**M**). The differences between the various groups were determined using a one-way analysis of variance. The data are expressed as the mean ± SD (*n* = 3 for each group). * or ^&^ *p* < 0.05; ** *p* < 0.01; *** or ^&&&^ *p* < 0.001; ns: not significant.

We measured the 661 w cell viability in vitro using the CCK8 assay with treatments of different glucose concentrations. The data are shown in Figure 3A. The viability of the 661 w cells was decreased after high-glucose treatment for 24 h and 48 h compared to the NC group (*p* < 0.01, *p* < 0.001). Subsequently, we examined the apoptosis in the 661 w cells after high-glucose treatment. As shown in Figure 3B–D, the expression of apoptosis-related protein Bcl2 was decreased and Bax expression was increased after high-glucose treatment compared to the control group (*p* < 0.05, *p* < 0.01). We also evaluated the O-GlcNAc modification changes. The Western blot results show that the expressions of O-GlcNAc and OGT were increased and OGA expression was decreased after high-glucose treatment compared to the control group (*p* < 0.05, *p* < 0.01, *p* < 0.001) (Figure 3E–H). Mannitol was also used to treat the cells, confirming the osmotic pressure effect in the experiments. The micro-environment is also important for the cells, so we also used the conditioned medium (CM1/CM2) from Müller-Lacz/Müller-Trx cells, whose Trx stable overexpression can affect Müller cell function in the high-glucose condition, to treat the 661 w cells, aiming to explore the effect of the micro-environment on photoreceptor cells in DR. TUNEL staining showed that apoptosis was decreased and the O-GlcNAc modification changes were also decreased after CM1 or CM2 treatment under high-glucose treatment. The results suggest that 661 w cell apoptosis may be associated with O-GlcNAc modification changes after high glucose treatment.

### 2.4. AMPK Activation Affects 661 W Cell Apoptosis via O-GlcNAc Modification Changes after High Glucose Treatment

**Figure 4:**
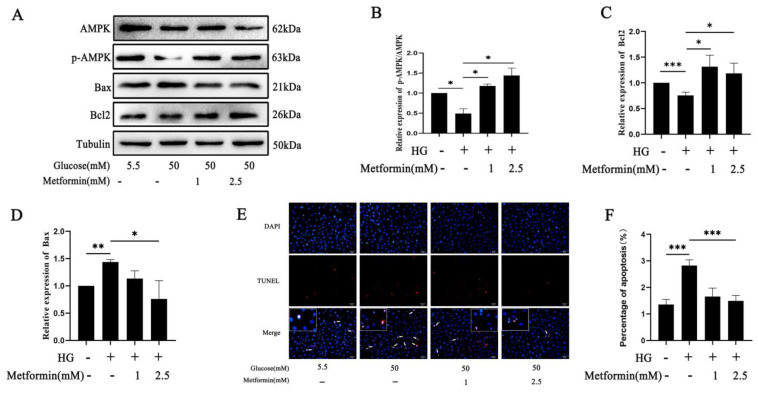

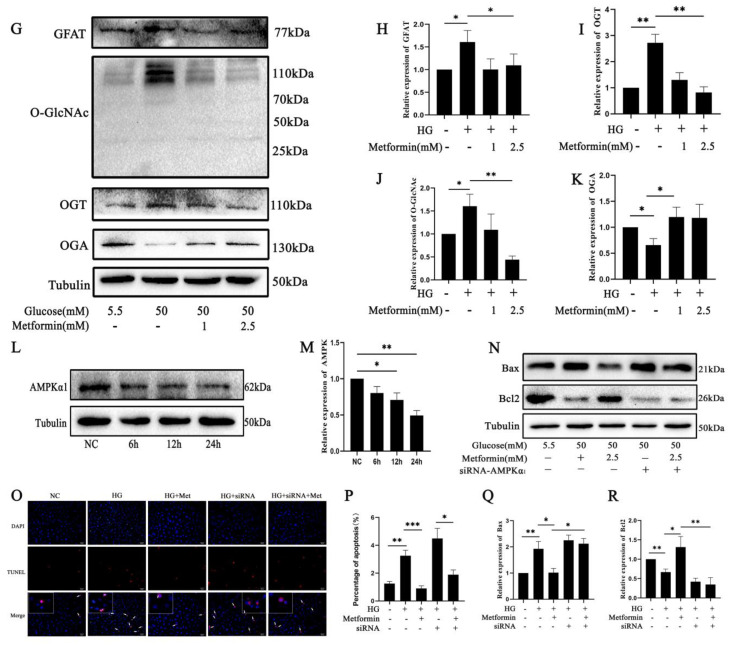
AMPK activation delayed 661 w cell apoptosis by regulating O-GlcNAc modification after high glucose treatment. (**A**) AMPK activation affected the expression of Bax, Bcl2, and p-AMPK in 661 w cells after high-glucose treatment. Western blot band analysis of p-AMPK/AMPK (**B**), Bax (**C**), and Bcl2 (**D**). (**E**) Effects of AMPK activation on high-glucose-induced apoptosis in 661 w cells detected using TUNEL staining. The arrows pointed the positive cells. The scale bar is 50 μm. (**F**) Statistical analysis of apoptosis using TUNEL staining. (**G**) Effects of AMPK activation on O-GlcNAc modification changes in 661 w cells after high-glucose treatment detected using Western blot. Statistical analysis of GFAT (**H**), OGT (**I**), O-GlcNAc (**J**), and OGA (**K**) was carried out using Western blot. (**L**) The expression of AMPKα1 was detected using Western blot after siRNA treatment at different times. (**M**) Representative Western blot analysis of AMPKα1. (**N**) AMPKα1 affected the expression of Bax and Bcl2 in 661 w cells after high-glucose treatment. (**O**) Effects of AMPKα1 on apoptosis in 661 w cells after high-glucose treatment were detected using TUNEL staining. The arrows pointed the positive cells. The scale bar is 50 μm. (**P**) Statistical analysis of apoptosis using TUNEL staining. (**Q**) Representative Western blot statistical analysis of Bax. (**R**) Representative Western blot statistical analysis of Bcl2. The differences between the various groups were determined using a one-way analysis of variance. The data are expressed as the mean ± SD (*n* = 3 for each group). * *p* < 0.05; ** *p* < 0.01; *** *p* < 0.001.

We used TUNEL staining and detected apoptotic-related protein expression in vitro using Western blot to evaluate the effects of AMPK activation on photoreceptor cell apoptosis in diabetic retinopathy. Bcl2 expression was decreased and Bax expression was increased after high-glucose treatment compared to the control group, which was reversed after AMPK activation (*p* < 0.05, *p* < 0.01) (Figure 4A–D). Moreover, the results show that the number of apoptotic positive cells was significantly increased in the 661 w cells after high-glucose treatment compared to the control group, while it was decreased after AMPK activation using different concentrations of metformin treatment (*p* < 0.001) (Figure 4E,F). We also evaluated the O-GlcNAc modification changes after AMPK activation. The Western blot analysis showed that O-GlcNAc expression and OGT expression were significantly increased; however, OGA expression was decreased, which was reversed after AMPK activation (*p* < 0.05, *p* < 0.01) (Figure 4G–K). These results indicate that AMPK activation regulates O-GlcNAc modification to delay photoreceptor cell apoptosis in diabetic retinopathy in vitro.

We used the siRNA method to downregulate AMPKα1 subunit expression. The results show that AMPKα1 expression was decreased after transfection with the AMPKα1 siRNA sequences at different times. Based on the experimental results, we confirmed the treatment time of the AMPKα1 siRNA sequence was 24 h (*p* < 0.01) (Figure 4L,M). Subsequently, we used TUNEL staining and Western blot to detect related proteins to evaluate the apoptosis. The results show that the number of apoptotic positive cells was increased in the 661 w cells after high-glucose treatment compared to the NC group, and the apoptotic cell number was decreased after AMPK activation. However, it was reversed after AMPKα1 downregulation (*p* < 0.05, *p* < 0.01, *p* < 0.001) (Figure 4N–R). These results suggest that the AMPKα1 subunit plays a vital role in activating AMPK to attenuate photoreceptor cell apoptosis in diabetic retinopathy in vitro.

### 2.5. Effects of Attenuating 661 W Cell Apoptosis on Neovascularization during High Glucose Treatment In Vitro

**Figure 5:**
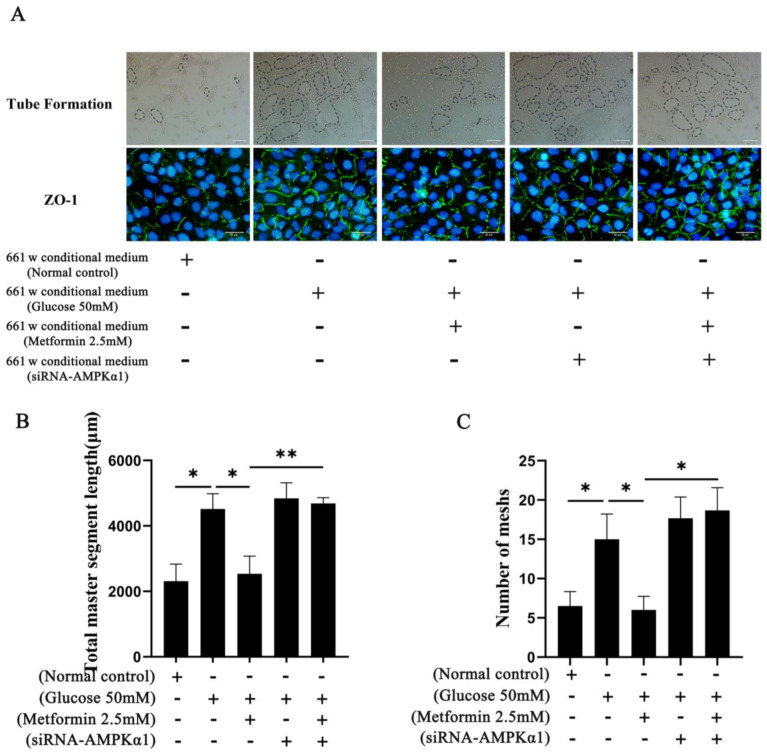
Effects of attenuating 661 w cell apoptosis on neovascularization after high glucose treatment in vitro. (**A**) Evaluation of the tube formation ability and detection of the distribution of the tight junction protein ZO-1(green color) in HUVEC after different treatments with the conditional medium of 661 w cells. The scale bar is 100 μm and 20 μm. (**B**) Statistical analysis on the number of total master segment lengths in HUVEC. (**C**) Statistical analysis of the number of meshes in HUVEC. The differences between the various groups were determined using a one-way analysis of variance. The data are expressed as the mean ± SD (*n* = 3 for each group). * *p* < 0.05; ** *p* < 0.01.

We used a tube formation assay and an immunofluorescence staining assay to evaluate the angiogenic state of blood vessels in high-glucose environments. The angiogenesis experiments showed that the number of meshes formed and the total master segment length were increased compared to the control group after the 661 w cells were treated with a conditional medium. However, the process was inhibited after AMPK activation in the 661 w cells, and the process was reversed after downregulating AMPKα1 in the 661 w cells (*p* < 0.05) (Figure 5A–C). We also detected the distribution of ZO-1 using immunofluorescence in HUVEC after the different treatments. The results show that the immunofluorescence intensity and distribution of ZO-1 were increased compared to the NC group in HUVEC after the 661 w cells were treated with a conditional medium. However, the expression was decreased and the distribution was irregular after AMPK activation in the 661 w cells, and the process was reversed after down-regulating AMPKα1 in the 661 w cells (Figure 5A).

### 2.6. AMPK Activation Attenuates the Expression of GFAT and TXNIP-O-GlcNAc Modification Changes in 661 W Cell Apoptosis after High Glucose Treatment In Vitro

**Figure 6:**
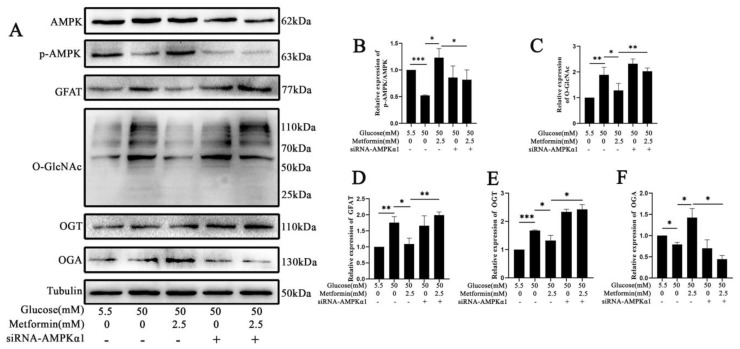

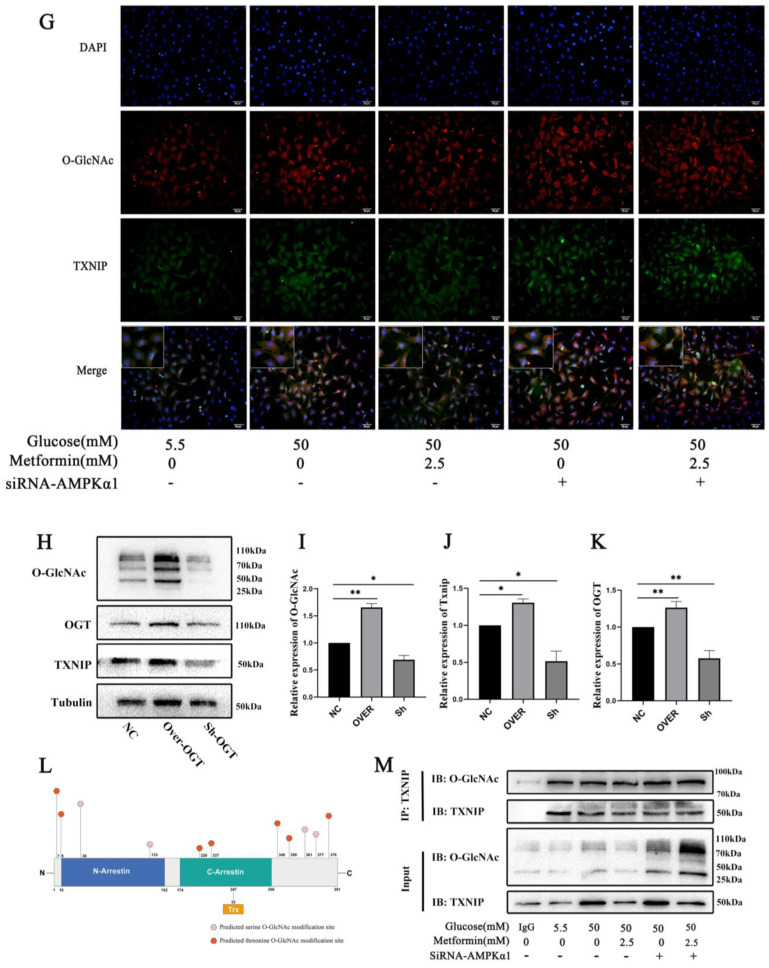

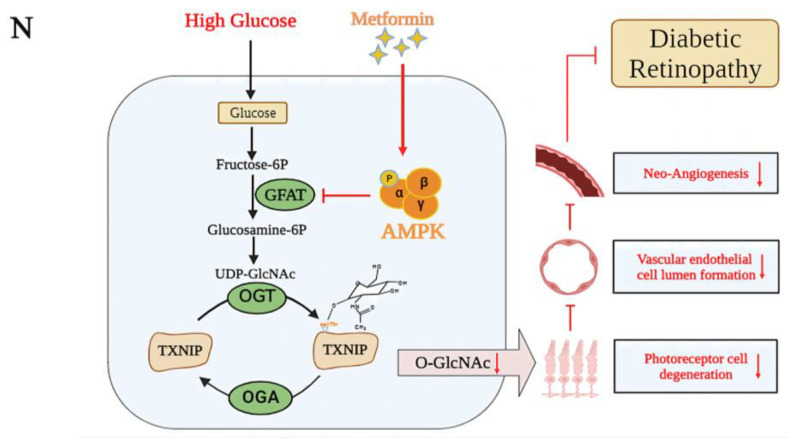
AMPK activation attenuated the expression of GFAT and O-GlcNAc modification changes in diabetic retinopathy-induced photoreceptor cell apoptosis in vitro. (**A**) Western blot was used to detect the expressions of p-AMPK, GFAT, and O-GlcNAc modification in 661 w cells after high-glucose treatment with or without AMPK activation. Statistical analysis of the expressions of p-AMPK (**B**), O-GlcNAc (**C**), GFAT (**D**), OGT (**E**), and OGA (**F**). (**G**) The co-expression of O-GlcNAc(red color) and TXNIP(green color) in 661 w cells after high-glucose treatment with and without AMPK activation was detected by using immunofluorescence staining. The scale bar is 50 μm. (**H**) Western blot was used to detect TXNIP expression after the upregulation and downregulation of O-GlcNAc modification changes in 661 w cells. Statistical analysis of the expressions of O-GlcNAc (**I**), TXNIP (**J**), and OGT (**K**). (**L**) O-GlcNAc modification site prediction for TXNIP. (**M**) The expressions of TXNIP and O-GlcNAc modification changes in 661 w cells after activation and inactivation of AMPK in high-glucose treatment were examined using a CO-IP assay. (**N**) A hypothetical working model of the molecular regulatory mechanism of AMPK activation on O-GlcNAc modification to delay diabetic retinopathy. The differences between the various groups were determined using a one-way analysis of variance. The data are expressed as the mean ± SD (*n* = 3 for each group). * *p* < 0.05; ** *p* < 0.01; *** *p* < 0.001.

Aiming to explore the underlying mechanism during the process, we detected the expression of signaling pathway proteins. In Figure 6A–F, the results show that in the AMPK activation intervention group, the expressions of GFAT and O-GlcNAc modification were increased after high-glucose treatment (*p* < 0.05, *p* < 0.01). However, the expressions of GFAT and O-GlcNAc modification were increased after silencing the AMPKα1 subunit (*p* < 0.05, *p* < 0.01). Furthermore, we also used immunofluorescence staining to detect the co-locations of TXNIP and O-GlcNAc and explore the O-GlcNAc protein. In Figure 6G, the results show that TXNIP and O-GlcNAc co-localize in the cytoplasm. The fluorescence of TXNIP and O-GlcNAc was increased after high-glucose treatment, but AMPK activation can reverse this process. However, the expressions of GFAT and O-GlcNAc modification were increased after silencing the AMPKα1 subunit. These results indicate that the mechanism by which AMPK activation attenuates 661 w cell apoptosis after high glucose treatment is related to GFAT/TXNIP-O-GlcNAc modification changes.

To further confirm the O-GlcNAc modification changes on TXNIP, we upregulated and downregulated the O-GlcNAc modification by overexpressing and knocking down the level of OGT, respectively, and observed the alteration in TXNIP expression in this process. The Western blot results are shown in Figure 6H–K (*p* < 0.05, *p* < 0.01). Furthermore, we also used http://services.healthtech.dtu.dk/ accessed on 13 March 2023 to predict the potential O-GlcNAc modification sites for TXNIP existence, and the results indicate 11 potential binding sites (Figure 6L). Finally, we verified the relationship between TXNIP and O-GlcNAc using a CO-IP assay to confirm the TXNIP-O-GlcNAc modification that appeared in this process (Figure 6M). All the results are summarized in Figure 6N.

## 3. Discussion

Diabetic retinopathy (DR) is one of the most serious diabetes complications and one of the main causes of visual impairment and blindness in the global working-age population [4]. DR is mainly clinically diagnosed and classified depending on whether retinal microvascular lesions are observed in an ophthalmic fundus examination [28]. However, this generally occurs at the late stage of DR, and the effect of treatment is limited for patients. Until now, none of the treatments used have fully attenuated clinical progression or reversed damage to the retina. Therefore, discovering new molecular targets is important to delay and prevent blindness caused by DR. Despite there are many studies focusing on DR, the complexity of DR pathogenesis is not fully understood until now.

In DR, hyperglycemia leads to an increased flux of the hexosamine synthesis pathway, which leads to an increase in the generation of its end product, UDP-GlcNAc. This acts as a substrate for O-GlcNAc, which is increasingly catalyzed by OGT. Some studies have reported that O-GlcNAc plays a key role in many pathological and physiological processes [29]. Due to its possible diversity of action, the dysregulation of O-GlcNAc is associated with a variety of disease developments, such as diabetes [30], diabetic complications [31], cancer [32], and neurodegenerative diseases [33].

In our study, it was found that 661 w cell apoptosis and photoreceptor cell degeneration in diabetic mice were associated with O-GlcNAc modification changes. The results show that the apoptosis of 661 w cells was increased after high-glucose treatment. Additionally, OGT expression was increased, and the expression of OGA was decreased. This suggests that O-GlcNAc modification is related to photoreceptor cell degeneration in DR. Furthermore, we also evaluated the effects of micro-environment changes on the O-GlcNAc modification in 661 w cells. These findings suggest that O-GlcNAc modification could be a promising target for the prevention or treatment of DR in the future.

Based on the data we obtained in this study, O-GlcNAc is related to DR; therefore, we need to explore an effective method to intervene. The results show that O-GlcNAc modification and diabetes-induced photoreceptor cell degeneration decreased after AMPK activation during DR. However, our study showed that AMPK activation was inhibited by compound C, and the thickness of the retina and ONL were also reduced. Compound C is a widely used ATP-competitive AMPK inhibitor, but there could be a lack of target selectivity which could affect the efficiency of AMPK activation inhibiting. In this manner, the researchers can also consider other inhibitors of AMPK such as SU6656, BAY-3827, MT 47–100, etc. AMPK exists as a heterotrimeric complex consisting of an α-catalytic subunit, a β-regulatory subunit, and a γ-regulatory subunit. The catalytic role of the α-subunit has two different isoforms (α1, α2) [34]. In our study, when silencing AMPKα1, the previous activation of AMPK was reversed, and the level of O-GlcNAc modification was increased again in the high-glucose environment. Apoptotic protein Bax expression was increased, and anti-apoptotic protein Bcl2 expression was decreased. This indicates that the AMPKα1 subunit plays an important role in this process.

Neovascularization is also another very important sign of DR in the clinic. The retina is very complex and consists of retinal pigment epithelium cells, photoreceptors, horizontal and bipolar cells, amacrine cells, ganglion cells, Müller cells, astrocytes, microglia, and blood cells. The relationships among these cell types could be taken into consideration. Other researchers have also mentioned that the retinal neurovascular unit could be a key element in understanding the retinal dysfunction that occurs in diabetic retinopathy [35,36]. Therefore, we also evaluated the effects of neurodegeneration inhibition on neovascularization in vitro under high-glucose conditions. Aiming to explore neovascularization in DR, we selected human umbilical vein endothelial cells (HUVECs) as the model to treat with conditional medium from 661 w cells. The results show that the number of tube formations in the HUVECs was significantly increased after high-glucose treatment; however, it was reduced after 661 w cell degeneration inhibition, decreasing with the O-GlcNAc modification changes via AMPK activation. We also detected a cellular tight junction structure in the HUVECs. The results show that the tight junctional structure distribution among the HUVECs was more complete after the 661 w cell high-glucose treatment, which was reversed after the AMPK activation treatment in the 661 w cells. This suggests that retinal neurodegeneration inhibition can reduce neovascularization in DR in vitro. Therefore, reducing neovascularization through O-GlcNAc modification changes in the photoreceptor cells could also become a promising therapeutic approach to delay the development of DR in the future.

Aiming to further explore the underlying mechanism, under the high glucose treatment, we found that the expression of GFAT was downregulated after AMPK activation. Additionally, the expressions of TXNIP and O-GlcNAc modification were also decreased under high glucose treatment after AMPK activation. This suggests that downregulating the GFAT/TXNIP-O-GlcNAc modification signaling axis inhibits DR through AMPK activation. Another study also reported that inhibiting retinal protein O-GlcNAcylation can delay neovascularization in DR [37]. However, the O-GlcNAc modification of the specific protein is not fully understood. Therefore, it could be a promising method to find the specific protein O-GlcNAc modification as a target that will provide help for the treatment of DR patients. In our study, we confirmed that TXNIP-O-GlcNAc modification happened in this process. Based on the available studies, it is known that AMPK, as a key molecule, regulates biological energy metabolism and is at the center of research on diabetes and other metabolism-related diseases [21]. There are various metabolism-related organs that express AMPK, which can be activated by various types of stimulation in the body, such as hormones, cellular stress, exercise, and substances that can also affect cellular metabolism [21]. Some studies have also found that AMPK activation has a protective effect against disease [38,39]. In our research, some results showed that AMPK activation can delay DR, which is consistent with the reported research that AMPK plays an important role in diabetes complications, but in our study, the related research focus and molecular mechanism are somewhat different from those of previous studies.

In summary, our study highlights the role of O-GlcNAc modification changes in DR and the reduction in photoreceptor cell degeneration by activating AMPK to decrease neovascularization in vitro. This work provides novel insights into the mechanisms linked to downregulating the GFAT/TXNIP-O-GlcNAc modification signaling axis. In addition, we also explored the more likely key role of the AMPKα1 subunit in the whole intervention process. These findings will provide new evidence for possible therapeutic targets for DR. Additional studies are required to identify O-GlcNAc modification as a promising therapeutic approach in diabetic retinopathy in the future.

## 4. Materials and Methods

### 4.1. Bioinformatics Analysis

In this study, the GEO database was used to analyze the differential expressions of OGT and OGA genes in groups of DR patients and normal people (source of the dataset: GSE102485 which contains the sample information, differential gene, and its expression) and to assess their predictive value for clinical diagnosis. We included 30 diabetic patients and 30 normal people. All patients were recruited from the First and Second Affiliated Hospital of Dalian Medical University (2020NO.008 and PJ-KS-KY-2023-450). Blood glucose measurements and blood samples (serum) were taken from diabetic patients and normal people. The concentrations of OGT and OGA were detected in the serum samples with the ELISA kit (Kexing, Shanghai, China).

### 4.2. Animal

C57BL/6 mice (female and male, 4~6 weeks, 20~25 g) were purchased from the Dalian Medical University Animal Experiment Center. The mice were acclimatized and fed for one week for adaptive feeding-free access to food and water and then selected to induce a diabetic mouse model. The mice were intraperitoneally injected with STZ (50 mg/kg) after 12 h of fasting once a day; this process was carried out for 5 consecutive days [40]. The normal control group was intraperitoneally injected with an equal volume of sodium citrate buffer. As the random blood glucose was more than 16.7 mmol/L, the model is considered to have been successfully constructed [11]. The entire experimental protocol was approved by the Institutional Animal Care and Use Committee of the Dalian Medical University Laboratory Animal Center (L20120021 and AEE22090). The experimental mice were randomly divided into a normal control group (NC), diabetic group (DM), diabetes with metformin treatment group (DM + Met), and diabetes with metformin and AMPK pathway inhibitor compound C treatment group (DM + Met + CC) (*n* = 6). The metformin was injected at a dose of 100 mg/kg, and compound C (Med Chem Express, Monmouth Junction, NJ, USA) was injected at a dose of 0.1 mg/kg for 7 days. The mice were executed after treatment under all experimental conditions.

### 4.3. Cell Culture and Reagents

Professor Muayyad R. AI-Ubaidi from the University of Oklahoma Health Sciences Center provided the 661 w cell line (the mouse photoreceptor-derived cell line). The 661 w cells were cultured in Dulbecco’s modified Eagle’s medium (DMEM, Gibco, 1 g/L D-glucose) containing 10% fetal bovine serum (FBS) and 1% antibiotic–antimycotic solution (penicillin/streptomycin) at 5% CO_2_ and 37 °C in a humidified incubator. The medium was replaced every 1 or 2 days. Human umbilical vein endothelial cells (HUVECs) were cultured in Dulbecco’s modified Eagle’s medium (DMEM, Gibco, 4.5 g/L D-glucose) containing 10% fetal bovine serum (FBS), 1% antibiotic–antimycotic solution (penicillin/streptomycin) at 5% CO_2_ and 37◦C in a humidified incubator. The medium was replaced every 1 or 2 days. Metformin (Sigma-Aldrich, Beijing, China) was dissolved in PBS as a 50 mM concentration stock solution and stored at −20 °C in darkness. The concentrations of metformin used in the experiment were 1 mM and 2.5 mM for 24 h.

### 4.4. Hematoxylin–Eosin Staining and Morphology Analysis

After the mice were sacrificed, their eyes were removed. The eyeballs were fixed for 24 h in Bouin’s regent and then stored for 1~3 days in 75% ethanol. After dehydration in different concentrations of ethanol, the samples were embedded in paraffin and then cut into sections with a thickness of 5 μm. The sections, which contained the optic nerve, were stained with hematoxylin and eosin (H&E) for morphology analysis. The total retina and outer nuclear layer (ONL) were observed under a microscope, and their thicknesses were measured using the software Nikon ECLIPSE 80i (Ver5.30.00).

### 4.5. Electroretinography (ERG)

ERG detection was performed with an electrophysiology instrument and system (Guote Medical, V8.1, Chongqing, China). The experimental mice were placed in the dark overnight to adapt before the experiment. The mice were placed under deep anesthesia by intraperitoneally injecting pentobarbital sodium (75 mg/kg), and 0.5% tropine and 0.5% phenylephrine were used to dilate the pupils. The anesthetized mice were placed on the detection platform and properly connected to the electrodes according to the operating instructions. The amplitudes of the a and b waves were detected and recorded using the 3 cd.s/m^2^ flash intensity after light stimulation in the following experimental groups.

### 4.6. Cell Viability Assay

The Cell Counting Kit-8 (CCK-8, Bioss, Beijing, China) assay was used to measure the cell viability. A total of 3 × 10^3^ cells were seeded into each well of a 96-well plate (Guangzhou Jet Bio-Filtration Co., Ltd., Guangzhou, China) with different concentrations (10 mM, 30 mM, 50 mM) of glucose for treatments with different time gradients. After treatment, 10 μL of CCK-8 reagent was added to each well and then incubated at 37 °C for 1~2 h in the darkness. The absorbance for each well was determined using a microplate reader at a wavelength of 450 nm (Varioskan Flash, Thermo Fisher, Waltham, MA, USA).

### 4.7. Immunofluorescence Staining

The cells were seeded on a cover slide in a 24-well plate with different treatments. Then, the cells were fixed for 20 min at room temperature in a 4% cell fixative solution. After washing with 0.025% TBST, the cover slides were incubated with 0.25% TritionX-100 for 30 min at room temperature and blocked with 0.1% TBST containing 10% normal horse serum at room temperature for 1 h before incubating with primary antibodies at 4 °C overnight. The following primary antibodies were used: anti-O-GlcNAc (abcam, ab2739, 1:200, RRID: AB_303264) and anti-TXNIP (Proteintech, Wuhan, China, 18243-1-AP, 1:200, RRID:AB_2876873). After washing three times with 0.025% TBST, the cover slides were incubated with secondary antibodies (Alexa Fluor 488, Invitrogen, A-11034, 1:1000, RRID: AB_2576217, and Dylight 594, Abbkine, A23610-1, 1:1000) for 1 h at room temperature in the dark. After washing with 0.025% TBST 3 times for 3 min, the nuclei were stained with DAPI regent solution for 15 min and photographed using fluorescence microscopy (Leica DM6000B fluorescence microscope, Wetzlar, Germany). For taking pictures, at least three different fields were randomly selected for observation in each slide from different groups. The condition for taking pictures was setting up the same for every experimental group.

### 4.8. Western Blot

The samples were collected and lysed on ice in a tube with an appropriate amount of lysis solution (Keygan, KGP2100). The samples were sonicated, incubated for 30 min on ice, and centrifuged with 12,000× *g* for 15 min at 4 °C. The protein concentration was measured using the BCA assay (Keygan, KGP903). The equal quantities of proteins were separated by sodium dodecyl sulfate-polyacrylamide gels (SDS-PAGEs) and transferred to a polyvinylidene fluoride (PVDF) membrane (Immobilon-PSQ, ISE00010, Millipore). The membranes were blocked with 5% non-fat milk for an hour at room temperature. The membranes were incubated with anti-Tubulin (Proteintech, 66031-1-Ig, 1:10,000, RRID: AB_11042766), anti-O-GlcNAc (Abcam, ab2739, 1:1000, RRID: AB_303264), anti-OGT (Proteintech, 11576-2-AP, 1:1000, RRID: AB_2156943), anti-OGA (Proteintech, 14711-1-AP, 1:1000, RRID: AB_2143063), anti-Bax (Proteintech, 60267-1-Ig 1:5000, RRID:AB_2848213), anti-Bcl2 (Abmart, T40056, 1:1000, RRID:AB_2929011), anti-AMPK (Proteintech, 66536-1-Ig, 1:1000, RRID: AB_2881899), anti-p-AMPK (Cell Signaling, 2535, 1:1000, RRID: AB_331250), and anti-GFAT (Proteintech, 14132-1-AP, 1:1000, RRID: AB_2110155) antibodies overnight at 4 °C. After washing 3 times with 1 × TBST for 15 min each, the membranes were incubated with goat anti-mouse IgG (Proteintech, SA00001-1, 1:5000, RRID: AB_2722565) and goat anti-rabbit IgG (Proteintech, SA00001-2, 1:5000, RRID: AB_2722564) secondary antibodies for 1.5 h at room temperature, and then washed again as described above. The protein bands were visualized using an enhanced chemiluminescence system, and the band intensities were analyzed using Image Lab software (Version:4.1.0.2177) and Image J software (Version:2.0.0-rc-43/1.50e). The relative expressions were normalized using an internal control (Tubulin).

### 4.9. TUNEL Staining

The samples were fixed with 4% paraformaldehyde at room temperature for 20 min. Staining was performed according to the TUNEL assay (Keygan, KGA7061) instructions. The nuclei were stained with DAPI regent solution for 15 min and photographed using fluorescence microscopy (Leica DM6000B fluorescence microscope, Germany).

### 4.10. Tube Formation Assay

The tube formation ability of human umbilical vein endothelial cells (HUVECs) was evaluated using Matrigel (Yensen, Wuhan, China, 40184ES08). Before the experiment, the gels were allowed to polymerize in 24-well plates (100 μL gels per well) for 30 min in a 37 °C incubator. The HUVECs were seeded at 8 × 10^4^ cells per well for 24 h in a 37 °C, 5% CO_2_ incubator with medium for different conditions. After washing with PBS, tube formation was observed under a light microscope. Images were captured with three different fields in each group. To quantify the tube formation, the number, area, and length of the formed tubes were calculated using Image J software (Version:2.0.0-rc-43/1.50e).

### 4.11. CO-IP Assay

The cells were lysed and incubated with the primary antibody overnight at 4 °C, and then 30 μL Protein A/G Plus-Agarose (Proteintech, Wuhan, China) was added. The samples were mixed on a rocker platform at 4 °C for 4~6 h and then centrifuged at 10,000 rpm for 2 min at 4 °C. The magnetic beads were washed and resuspended in an Elution buffer. Then, the samples were boiled for 5 min to release the proteins and centrifuged to remove the Protein A/G beads. Finally, the complexes were tested using Western blot.

### 4.12. Statistical Analysis

The differences between the two groups were determined using a *t*-test, and the various groups were determined using a one-way analysis of variance. All data are presented in this study as the mean ± SD. A *p* value < 0.05 is considered statistically significant.

## Data Availability

Data are available upon request.
